# Astragaloside IV attenuates post-ischemic neuroinflammation associated with Treg-related regulation of CD86/CD206-associated brain myeloid phenotypic responses and PI3K/AKT signaling alterations

**DOI:** 10.3389/fimmu.2026.1939189

**Published:** 2026-10-01

**Authors:** Hengtong An, Shuhan Guo, Shaoze Yang, Tianci Zhang, Xiaowen Li, Zejun Ma, Yitong Wang, Ruotong Wang, Xiaohong Zhou, Weijuan Gao

**Affiliations:** 1 Hebei University of Chinese Medicine, Shijiazhuang, China; 2 Hebei Key Laboratory of Chinese Medicine Research on Cardio-Cerebrovascular Disease, Hebei University of Chinese Medicine, Shijiazhuang, China

**Keywords:** astragaloside IV, brain myeloid cells, ischemic stroke, neuroinflammation, PI3K/AKT signaling, regulatory T cells

## Abstract

**Background:**

Ischemic stroke triggers persistent neuroinflammation, microglial activation, and peripheral immune changes that aggravate secondary brain injury. Astragaloside IV (AS-IV), a major bioactive constituent of *Astragalus membranaceus*, has neuroprotective and immunomodulatory effects, but its influence on Tregs and CD86/CD206-associated microglial phenotypic responses remains unclear.

**Aim:**

This study investigated whether AS-IV attenuates post-ischemic neuroinflammation in association with Treg-related immune responses, PI3K/AKT signaling alterations, and CD86/CD206-associated brain myeloid phenotypic responses.

**Materials and methods:**

A mouse middle cerebral artery occlusion (MCAO) model was established using the intraluminal filament method. Neurological deficits, infarct volume, and histopathological injury were evaluated after AS-IV treatment. Western blotting and ELISA were used to assess inflammatory cytokines and PI3K/AKT-associated signaling in ischemic brain tissue. Transcriptomic analysis identified AS-IV-related biological processes and pathways. Flow cytometry quantified CD4^+^CD25^+^FOXP3^+^ regulatory populations and additionally analyzed CD4^+^FOXP3^+^ populations in the spleen and cervical lymph nodes, together with CD86^+^/CD206^+^ phenotypic responses within the CD11b^+^CD45^low/int population in brain tissue. Immunofluorescence evaluated brain CD3^+^FOXP3^+^ signals and Iba1/CD86 or Iba1/CD206 double-positive signals. Anti-CD25 intervention was used to evaluate the involvement of Treg-related immune responses in AS-IV-associated effects.

**Results:**

AS-IV reduced infarct volume, showed a trend toward improvement in neurological deficit scores, and alleviated histopathological injury after MCAO. It decreased IL-6 and TNF-α and increased IL-10 and TGF-β in ischemic brain tissue. Transcriptomic analysis identified changes associated with the PI3K/AKT signaling pathway and immune regulatory processes, while western blotting showed increased p-PI3K/PI3K and p-AKT/AKT ratios after AS-IV treatment. AS-IV increased Treg proportions in the spleen and cervical lymph nodes and increased CD3^+^FOXP3^+^ signals in brain tissue. It also reduced CD86^+^-associated and increased CD206^+^-associated brain myeloid phenotypic responses. Anti-CD25 partially attenuated AS-IV-associated improvements in neurological and brain injury outcomes, as well as its effects on inflammatory cytokines and brain myeloid phenotypic responses.

**Conclusion:**

AS-IV attenuated post-ischemic neuroinflammation in association with Treg-related immune responses and PI3K/AKT-associated signaling alterations, accompanied by changes in CD86/CD206-associated brain myeloid phenotypic responses. These findings support AS-IV as a potential neuroimmunomodulatory intervention for ischemic stroke.

## 1 Introduction

Ischemic stroke is a major cause of mortality and long-term disability worldwide and remains a substantial public health burden (1). Its central pathological event is acute occlusion of a cerebral artery (2). Intravenous thrombolysis and endovascular thrombectomy have improved outcomes in selected patients, and endovascular indications and treatment windows continue to broaden. Even so, clinical benefit is constrained by time-dependent eligibility, patient selection, and secondary injury after reperfusion (3). Cerebral ischemia and reperfusion can trigger oxidative stress, blood–brain barrier disruption, immune cell infiltration, and sustained neuroinflammation, thereby amplifying neuronal damage and limiting neurological recovery (4). Strategies that restrain post-ischemic inflammation, reshape the neuroimmune microenvironment, and reduce neuronal injury therefore remain important in ischemic stroke research.

Neuroinflammation is closely involved in the evolution of ischemic brain injury. Following cerebral ischemia, microglia, the main resident immune cells of the central nervous system, sense local tissue damage and adopt diverse functional states. CD86-positive microglia are generally linked to pro-inflammatory responses and to the production of inflammatory cytokines, including interleukin-6 (IL-6) and tumor necrosis factor-alpha (TNF-α). By contrast, CD206-positive microglia are more often associated with inflammation resolution, tissue repair, and immunoregulatory activity (5). Increasing evidence has also drawn attention to crosstalk between brain-resident immunity and peripheral immune responses. Regulatory T cells (Tregs), especially FOXP3-positive Tregs, have been implicated in neuroimmune homeostasis (6). Prior studies suggest that brain-associated Tregs may restrain excessive adaptive immune activity and influence the post-injury inflammatory milieu by limiting glial overactivation, modulating cytokine release, and supporting reparative processes (7, 8). Treg-related immune regulation may therefore connect peripheral immune responses with microglial phenotypic responses in the brain. Whether pharmacological intervention can modulate this regulatory link and thereby relieve neuroinflammation after ischemic stroke remains unclear.

Astragaloside IV (AS-IV) is a naturally occurring cycloartane-type triterpenoid saponin and one of the major bioactive constituents of *Astragalus membranaceus*. Astragali Radix (Huangqi), the dried root of *Astragalus membranaceus*, has long been used in traditional Chinese medicine and is an important component of several classical prescriptions used for stroke-related disorders (9, 10). This traditional application, together with the established pharmacological activities of AS-IV, provides a rationale for investigating AS-IV in ischemic stroke. AS-IV has been associated with anti-inflammatory, antioxidant, anti-apoptotic, and immunomodulatory effects (11). Experimental studies indicate that AS-IV can protect against cerebral ischemic injury (12) and improve post-ischemic pathological changes by acting on inflammatory responses, oxidative stress, and cellular injury (13–15). The immune mechanisms underlying these effects, however, are not fully resolved. In particular, the relationship between AS-IV-associated Treg responses and post-ischemic neuroinflammatory regulation remains poorly understood.

Previous studies have already demonstrated that AS-IV can attenuate post-ischemic neuroinflammation and influence microglial phenotypic responses. Reductions in CD86 and pro-inflammatory cytokines, together with increases in CD206 and immunoregulatory mediators such as IL-10 and TGF-β, have been reported following AS-IV treatment (16, 17). These findings provide an important basis for understanding the anti-inflammatory effects of AS-IV after cerebral ischemia. However, whether these previously described neuroinflammatory and myeloid phenotypic responses are linked to Treg-related immune regulation remains insufficiently understood.

The PI3K/AKT signaling pathway participates in cell survival, inflammatory regulation, and immune homeostasis (18). During ischemic brain injury, PI3K/AKT-associated signaling has been linked to neuroprotection, inflammatory modulation, and cellular phenotypic responses. Recent evidence further indicates that AS-IV can modulate post-ischemic neuroinflammation through PI3K/Akt-related signaling, including the PI3K/Akt/NF-κB axis (19). However, how AS-IV-associated PI3K/AKT signaling changes relate to Treg-related immune responses and CD86/CD206-associated brain myeloid phenotypic responses remains incompletely understood.

Here, we used a mouse middle cerebral artery occlusion (MCAO) model to evaluate the effects of AS-IV on neurological deficits, cerebral infarct volume, histopathological injury, and inflammatory responses after ischemic stroke. Transcriptomic analysis, western blotting, enzyme-linked immunosorbent assay, flow cytometry, and immunofluorescence staining were then used to examine PI3K/AKT-associated signaling, Treg-related immune responses, and CD86/CD206-associated brain myeloid phenotypic responses. Anti-CD25 intervention was further introduced to test whether Treg-related immune regulation participates in the effects of AS-IV. Rather than re-establishing the anti-inflammatory, PI3K/AKT-associated, or CD86/CD206-related effects of AS-IV individually, the present study sought to integrate these responses within a broader neuroimmune framework and to determine whether Treg-related immune regulation may contribute to AS-IV-associated changes after MCAO. This approach extends previous work by placing Treg-related immune responses within the broader peripheral–central immune changes associated with AS-IV treatment after ischemic stroke.

## 2 Materials and methods

### 2.1 Animals and ethics

Male C57BL/6J mice of specific pathogen-free grade, weighing 22–25 g, were purchased from Henan Skobes Biotechnology Co., Ltd. All mice were acclimatized for 7 days before the experiments. All animal procedures were approved by the Ethics Committee of Hebei University of Chinese Medicine (approval No. KJLL(D)20251001) and were performed in accordance with institutional guidelines for the care and use of laboratory animals.

### 2.2 MCAO model and experimental design

The mouse middle cerebral artery occlusion (MCAO) model was generated using the intraluminal filament method. Briefly, mice were anesthetized with inhaled isoflurane and placed on a surgical platform. Isoflurane was used at approximately 3%–5% for induction and 1.5%–2.5% for maintenance. After cervical vessel exposure, the common carotid artery, external carotid artery, and internal carotid artery were isolated. A filament was advanced from the external carotid artery into the internal carotid artery to occlude the middle cerebral artery. After 1.5 h of ischemia, the filament was removed to initiate reperfusion. Mice in the Sham group underwent vascular exposure without filament insertion.

Mice were allocated to experimental groups using a random number table according to the purpose of each experiment. Neurological functional assessment and quantitative image analyses were performed by investigators blinded to group allocation. Successful MCAO modeling was assessed using the Zea–Longa neurological deficit score at 2 h after reperfusion, before initiation of post-MCAO treatment. Mice with scores of 1–4 were considered successfully modeled, whereas animals that failed to meet this predefined criterion were excluded before treatment. No formal *a priori* power calculation was performed; sample sizes were determined with reference to commonly used group sizes in previous MCAO studies, with additional animals included to account for expected perioperative mortality and unsuccessful modeling. Detailed information on animal allocation, perioperative mortality, exclusions due to failure of the neurological criterion, and the final number of successfully modeled animals is provided in **Supplementary Table S1**, while final sample sizes for individual experiments are reported in the corresponding figure legends.

The Sham, MCAO, AS-IV, and NBP groups were used for pharmacodynamic evaluation, whereas the Sham, MCAO, AS-IV, Anti-CD25, and Anti-CD25+AS-IV groups were used for mechanistic validation. Neurological outcomes were assessed 7 days after reperfusion as efficacy endpoints. Brain tissue, spleen, and cervical lymph nodes were then collected according to the experimental design. Brain tissue was used for TTC staining, hematoxylin and eosin (HE) staining, Nissl staining, western blotting, enzyme-linked immunosorbent assay (ELISA), RNA sequencing (RNA-seq), immunofluorescence staining, and flow cytometric analysis of brain myeloid phenotypic responses. Spleen and cervical lymph nodes were used mainly for flow cytometric analysis of Treg proportions.

### 2.3 Drug administration

AS-IV (Chengdu Must Bio-technology Co., Ltd.; catalog No. MUST-25033121; purity ≥98%) was initially dissolved with a small amount of DMSO and subsequently diluted with sterile 0.9% saline to a final concentration of 2 mg/mL, with a final DMSO concentration of 0.2%. The preparation was sonicated to facilitate dissolution and obtain a homogeneous formulation before administration. Mice in the AS-IV group received AS-IV beginning after MCAO reperfusion at a dose of 20 mg/kg by intraperitoneal injection once daily for 7 consecutive days. The injection volume was adjusted according to body weight at 10 mL/kg. Mice in the NBP group received butylphthalide (NBP; CSPC Pharmaceutical Group Limited; catalog No. 1182409206) at a dose of 90 mg/kg by oral gavage, with the same treatment duration and frequency as the AS-IV group.

To assess the involvement of Treg-related immune responses in the effects of AS-IV, mice in the Anti-CD25 group received anti-CD25 antibody intervention to attenuate Treg-related immune responses. The anti-CD25 antibody (clone PC61; BioLegend; catalog No. 102058) was administered intraperitoneally 2 days before MCAO at 250 μg/mouse. Mice in the Anti-CD25+AS-IV group received AS-IV treatment after MCAO reperfusion on the basis of anti-CD25 antibody intervention. Mice in the Sham and MCAO groups received equal volumes of vehicle containing 0.2% DMSO in sterile 0.9% saline.

### 2.4 Neurological function assessment

The Zea–Longa neurological deficit score was assessed at 2 h after reperfusion for MCAO model inclusion and again at 7 days after reperfusion as an efficacy outcome in the pharmacodynamic cohort. The 2-h assessment was performed before initiation of post-MCAO treatment and was used exclusively for model inclusion. The scoring criteria were as follows: 0, no obvious neurological deficit; 1, failure to fully extend the contralateral forelimb; 2, circling to the contralateral side; 3, falling toward the contralateral side; and 4, inability to walk spontaneously or impaired consciousness.

In the Anti-CD25 mechanistic cohort, neurological deficits were further evaluated using the 18-point modified Neurological Severity Score (mNSS) to provide a more comprehensive assessment of neurological function. Higher scores indicated more severe neurological impairment.

### 2.5 TTC staining and cerebral infarct volume analysis

Brain tissue was collected 7 days after reperfusion for TTC staining. Day 7 was selected as a unified subacute endpoint to allow neurological, histopathological, inflammatory, and immunological outcomes to be evaluated at the same stage after MCAO. After anesthesia and euthanasia, the brain was rapidly removed and briefly rinsed with pre-cooled phosphate-buffered saline (PBS). The whole brain was cut coronally into six slices of approximately 1 mm thickness. The brain slices were then incubated in TTC solution in the dark. After staining, the slices were fixed in 4% paraformaldehyde and photographed. Normal brain tissue appeared red, whereas infarcted tissue appeared white. ImageJ software was used to measure the infarct area and the areas of the ipsilateral and contralateral hemispheres. To minimize the effects of edema or tissue deformation, the corrected infarct area was calculated as: contralateral hemisphere area − (ipsilateral hemisphere area − infarct area). The percentage of corrected cerebral infarct volume was then calculated based on slice thickness for subsequent analysis.

### 2.6 HE and Nissl staining

For HE and Nissl staining, mice were transcardially perfused first with sterile saline and subsequently with 4% paraformaldehyde before brain collection. The brain tissue was then dehydrated, cleared, embedded, and cut into 4 μm sections. The sections were then subjected to HE staining and Nissl staining. After staining, the sections were observed under a light microscope. HE staining was used to evaluate brain tissue structure, cellular morphology, and ischemia-related pathological changes. Nissl staining was used to assess neuronal morphology and Nissl body changes. Nissl-positive neurons were counted in five fixed fields for each animal, and the mean value was used for statistical analysis.

### 2.7 RNA sequencing and bioinformatics analysis

Brain tissue was collected from the Sham, MCAO, and AS-IV groups on day 7 after MCAO for RNA sequencing, with three biological replicates in each group. Total RNA was extracted using TRIzol reagent. After quality assessment, library construction and paired-end RNA sequencing were performed by OE Biotech Co., Ltd. (Shanghai, China).

Raw sequencing data were quality-controlled using fastp v0.20.1, and adaptor sequences and low-quality reads were removed to obtain clean reads. The clean reads were aligned to the mouse reference genome Mus musculus NCBI GRCm39 (GCF_000001635.27) using HISAT2 v2.1.0. Gene expression quantification was performed using HTSeq-count v0.11.2 to obtain gene-level read counts, and fragments per kilobase of transcript per million mapped reads (FPKM) values were calculated to evaluate gene expression levels.

Differential expression analysis was performed using DESeq2 v1.22.2, mainly comparing the MCAO group with the Sham group and the AS-IV group with the MCAO group. Differentially expressed genes were identified using q-value < 0.05 and fold change > 1.5 for upregulated genes or fold change < 1/1.5 for downregulated genes as the cutoff criteria. For sample-level quality assessment, principal component analysis (PCA) was performed using log2(FPKM + 1)-transformed expression values across all nine RNA-seq samples. GO and KEGG pathway enrichment analyses were performed using a hypergeometric distribution-based approach, with the corresponding GO-annotated and KEGG-annotated background gene sets used as enrichment backgrounds. GO annotations were obtained from the Gene Ontology Resource and KEGG pathway annotations from the KEGG database, corresponding to the September 2023 update. P values were adjusted for multiple testing using the Benjamini–Hochberg method, and adjusted P values/q-values were reported in the enrichment results. To provide a less threshold-dependent assessment of pathway-level transcriptional changes, gene set enrichment analysis (GSEA) was additionally performed using the full ranked gene list without applying a predefined differential-expression fold-change cutoff. KEGG gene sets were used for pathway-level GSEA, and enrichment was evaluated using enrichment score (ES), normalized enrichment score (NES), nominal P value, and false discovery rate (FDR).

### 2.8 Western blotting

Ischemic hemisphere tissue was collected and lysed in RIPA lysis buffer supplemented with phosphatase and protease inhibitors to extract total protein. Protein concentration was determined using a BCA assay. Equal amounts of protein were separated by SDS-PAGE and transferred onto polyvinylidene fluoride (PVDF) membranes. After blocking, the membranes were incubated with primary antibodies overnight at 4 °C. The primary antibodies included IL-6 (1:1,000, Abcam, ab290735), IL-10 (1:1,000, Abcam, ab9969), TNF-α (1:1,000, Abcam, ab183218), TGF-β (1:1,000, Abclonal, A2124SP), PI3K (1:1,000, Abcam, ab191606), p-PI3K (1:1,000, Cell Signaling Technology, 4228), AKT (1:10,000, Abcam, ab179463), p-AKT (1:1,000, Proteintech, 66444-1-Ig), and β-actin (1:10,000, Proteintech, 81115-1-RR). Different target proteins were detected on separate gels and membranes rather than by stripping and sequential reprobing of the same membrane. Each target protein was analyzed with its corresponding β-actin loading control, and relative protein expression was calculated as the ratio of the target-protein band intensity to the corresponding β-actin band intensity. For phosphorylation analysis, phosphorylated and total protein signals were first normalized to their respective β-actin signals, and the ratios of the normalized phosphorylated to total protein values were then calculated. The membranes were then incubated with the corresponding secondary antibodies at room temperature for 2 h. After washing, the protein bands were visualized using chemiluminescence detection. Band intensities were measured using ImageJ software for statistical analysis.

### 2.9 ELISA

Ischemic hemisphere tissue was collected and homogenized with pre-cooled PBS. Protein concentration was determined using a BCA assay. The protein concentrations of IL-6, IL-10, TNF-α, and TGF-β in brain tissue were measured using corresponding ELISA kits. The kit information was as follows: IL-6 (Multi Sciences, EK206), IL-10 (Multi Sciences, EK210), TNF-α (Multi Sciences, EK282), and TGF-β (Multi Sciences, EK981). All procedures were performed according to the manufacturers’ instructions. The results were read as calibrated optical density (OD) values using a microplate reader with dual-wavelength measurement at 450 nm and 570 nm. The calibrated OD value was calculated by subtracting the 570 nm reading from the 450 nm reading, and the final concentration was calculated according to the standard curve. The results were expressed as pg/mg protein.

### 2.10 Flow cytometry

For flow cytometric analysis, mice were transcardially perfused with sterile saline before tissue collection. Spleen, cervical lymph nodes, and ischemic hemisphere tissue were then collected according to the experimental design. Single-cell suspensions from the spleen and cervical lymph nodes were prepared by mechanical dissociation and used for Treg detection. Tregs were detected using a Treg cell detection kit (Multi Sciences, KTR201). Cells were sequentially stained with the surface antibodies FITC-CD4 and APC-CD25 (clone PC61) and the intracellular antibody PE-FOXP3. Brain tissue was processed using a gentle enzymatic dissociation kit (RWD Life Science, DHABE-5003) together with a single-cell suspension preparation instrument. The prepared single-cell suspensions were used for brain myeloid phenotypic analysis. CD86 and CD206 were detected in separate staining tubes. Cells were stained with PE-CD11b and FITC-CD45 in combination with APC-CD86 or APC-CD206. The antibody information was as follows: CD45 (Selleck, G0016), CD86 (Selleck, G4413), CD206 (Selleck, G4068), and CD11b (Selleck, G4667).

Data were analyzed using CytExpert software. For Treg analysis, debris and cell aggregates were first excluded, and CD4^+^ cells were then gated. The proportion of CD25^+^FOXP3^+^ Tregs was finally analyzed within the gated CD4^+^ cell population. In addition to the conventional CD4^+^CD25^+^FOXP3^+^ phenotype, CD4^+^FOXP3^+^ regulatory populations were additionally analyzed independent of CD25 expression to minimize potential interference caused by anti-CD25 intervention. For brain myeloid cell analysis, debris and cell aggregates were first excluded, and CD11b^+^CD45^low/int cells were subsequently gated for phenotypic analysis. The proportions of CD86^+^ and CD206^+^ cells were analyzed within the gated CD11b^+^CD45^low/int population.

### 2.11 Immunofluorescence staining

For immunofluorescence analysis, separate animals were transcardially perfused first with sterile saline and subsequently with 4% paraformaldehyde before brain collection. After fixation and embedding, coronal brain sections were prepared. The sections were deparaffinized, rehydrated, subjected to antigen retrieval, and blocked. The sections were then incubated with primary antibodies overnight at 4 °C. The primary antibodies used for immunofluorescence staining included CD3, FOXP3, Iba1, CD86, and CD206. The antibody information was as follows: CD3 (Servicebio, GB11014-100), FOXP3 (Novus, NB100-39002), Iba1 (Novus, NB100-1028), CD86 (Boster, BM4121), and CD206 (Cell Signaling Technology, 24595). The sections were subsequently incubated with the corresponding fluorescent secondary antibodies, and nuclei were counterstained with DAPI. CD3/FOXP3 double immunofluorescence staining was performed to assess CD3^+^FOXP3^+^ signals in brain tissue, while Iba1/CD86 and Iba1/CD206 double staining was used to assess CD86- and CD206-associated brain myeloid phenotypic responses, respectively. For quantitative analysis, CD3^+^FOXP3^+^ cells were identified by nuclear FOXP3 staining associated with spatially corresponding CD3-positive membrane staining within the same cellular profile. Total cell number was determined based on DAPI-positive nuclei, and the proportion of CD3^+^FOXP3^+^ cells was calculated as the number of double-positive cells divided by the total number of DAPI-positive cells in each field. Iba1^+^CD86^+^ and Iba1^+^CD206^+^ double-positive areas were expressed as percentages of the corresponding Iba1^+^ area. Fluorescence images were acquired using a fluorescence microscope and quantitatively analyzed using ImageJ software.

### 2.12 Statistical analysis

All quantitative data were analyzed using GraphPad Prism 10.4.2. Continuous data are presented as mean ± SD, whereas Zea–Longa and mNSS neurological scores are presented as median with interquartile range (IQR). For continuous data satisfying the assumptions of parametric analysis and homogeneity of variance, comparisons among multiple groups were performed using one-way ANOVA followed by Tukey’s multiple-comparisons test. When the assumption of homogeneity of variance was not met, Welch’s ANOVA followed by Dunnett’s T3 multiple-comparisons test was used. Because the Zea–Longa and mNSS neurological scores are ordinal variables, comparisons among groups were performed using the Kruskal–Wallis test followed by Dunn’s multiple-comparisons test. A value of P < 0.05 was considered statistically significant.

## 3 Results

### 3.1 AS-IV attenuates ischemic brain injury after MCAO

To evaluate the protective effects of AS-IV in ischemic stroke, we established the MCAO model according to the experimental timeline in **Figure 1A** and then assessed infarct volume, neurological deficits, and histopathological injury. TTC staining revealed clear white infarct areas in the MCAO group, whereas AS-IV treatment markedly reduced the infarct area. The NBP group showed a comparable trend of improvement (**Figures 1B, C**). Neurological deficit scores were significantly increased after MCAO. AS-IV treatment showed a trend toward lower neurological deficit scores compared with the MCAO group; however, the difference did not reach statistical significance after Dunn’s multiple-comparisons correction (adjusted P = 0.0854; **Figure 1D**). HE staining showed intact tissue architecture and cellular morphology in the Sham group. In the MCAO group, tissue loosening, enlarged intercellular spaces, vacuole-like changes, and nuclear pyknosis were observed, and these pathological changes were alleviated by AS-IV treatment (**Figure 1E**). Nissl staining showed fewer Nissl-positive neurons after MCAO, whereas AS-IV treatment preserved more Nissl-positive neurons (**Figures 1F, G**). These findings indicate that AS-IV reduced cerebral infarct injury, showed a trend toward improvement in neurological deficits, and alleviated histopathological damage after MCAO.

**Figure 1:**
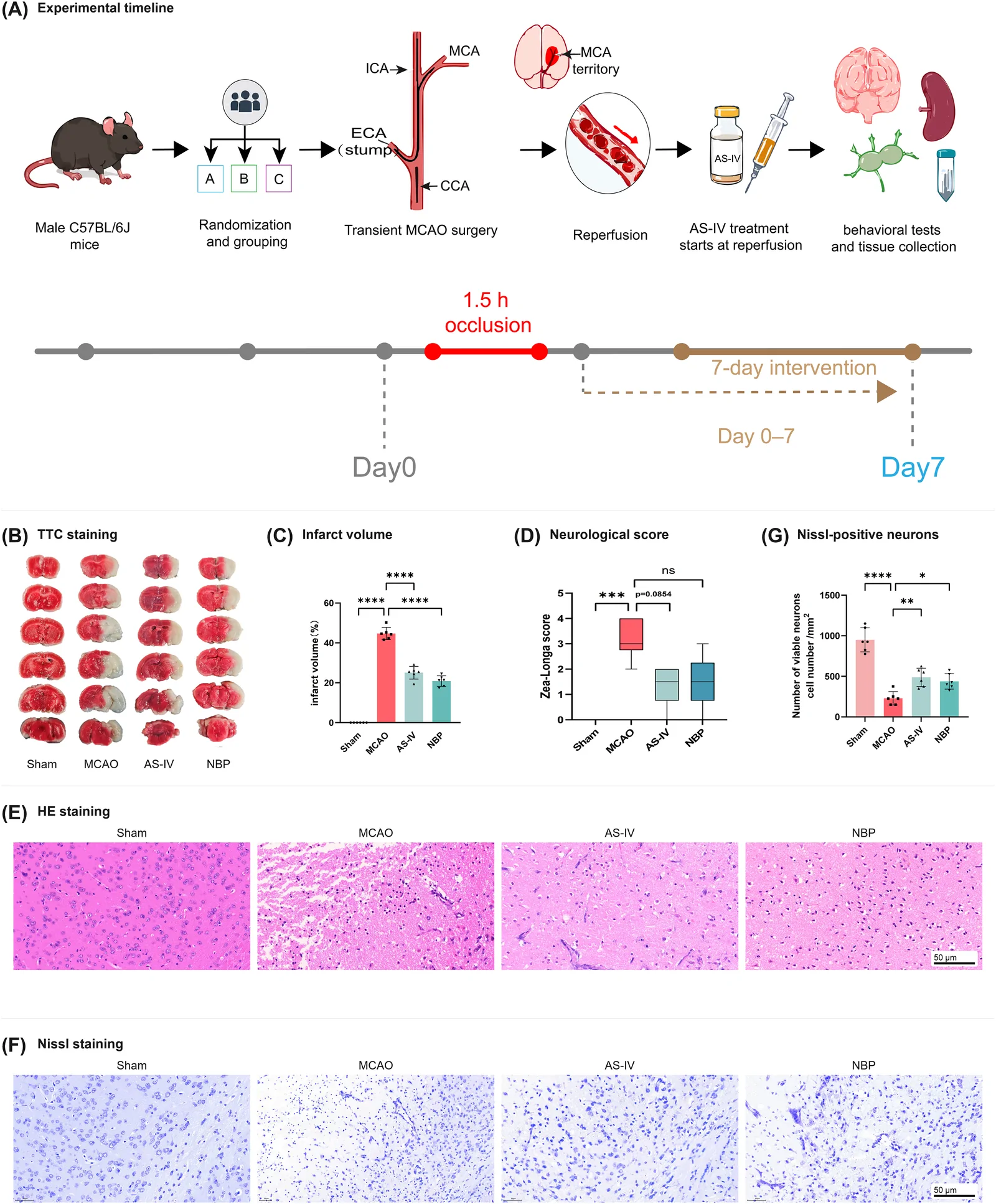
AS-IV reduces cerebral infarction and histopathological injury and shows a trend toward lower neurological deficit scores after MCAO. **(A)** Schematic illustration of the experimental timeline. **(B)** Representative TTC-stained brain sections from the Sham, MCAO, AS-IV, and NBP groups. **(C)** Quantification of cerebral infarct volume. **(D)** Zea–Longa neurological deficit scores. **(E)** Representative HE-stained brain sections. **(F)** Representative Nissl-stained brain sections. **(G)** Quantification of Nissl-positive neurons. Continuous data are presented as mean ± SD, whereas Zea–Longa neurological deficit scores in **(D)** are presented as median with interquartile range (IQR) (n = 6 mice per group). Brackets indicate comparisons between the indicated groups. *P < 0.05, **P < 0.01, ****P < 0.0001, ns indicates that the difference was not statistically significant.

### 3.2 AS-IV improves the neuroinflammatory microenvironment after MCAO

Western blotting and ELISA were next used to examine whether AS-IV altered the neuroinflammatory microenvironment after MCAO. Western blot analysis detected group differences in IL-6, TNF-α, IL-10, and TGF-β expression in brain tissue (**Figure 2A**). Quantification showed that IL-6 and TNF-α were increased in the MCAO group compared with the Sham group, whereas AS-IV reduced both pro-inflammatory cytokines relative to the MCAO group (**Figures 2B, C**). IL-10 and TGF-β expression increased after AS-IV treatment (**Figures 2D, E**). ELISA produced a similar pattern: AS-IV lowered IL-6 and TNF-α levels and increased IL-10 and TGF-β levels compared with the MCAO group. NBP intervention also showed an improving trend in inflammatory cytokine expression (**Figures 2F–I**). These results indicate that AS-IV improved the neuroinflammatory microenvironment by reducing pro-inflammatory cytokine levels and enhancing immunoregulatory cytokine expression.

**Figure 2:**
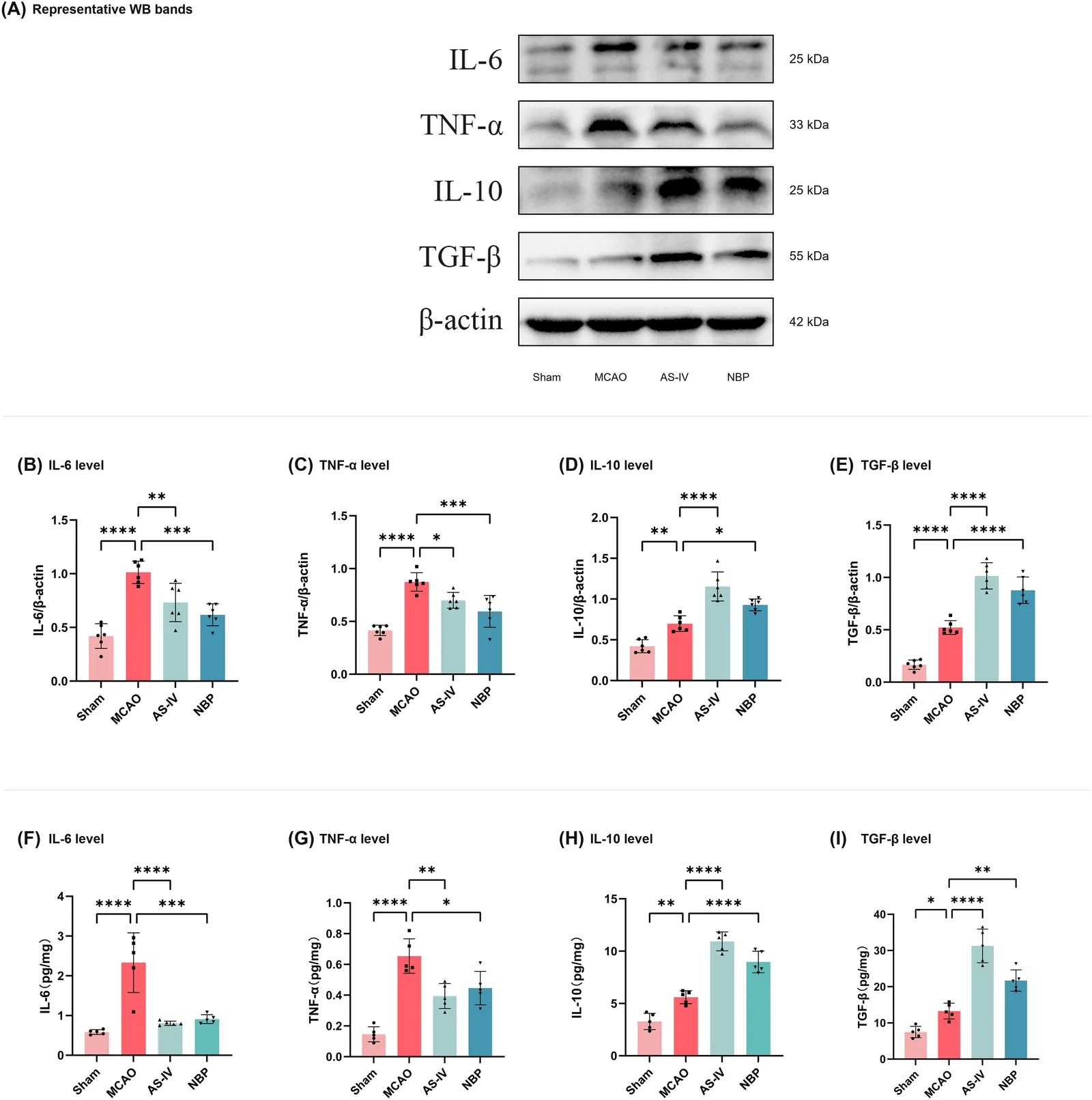
AS-IV modulates the inflammatory cytokine profile after MCAO. **(A)** Representative western blots of IL-6, TNF-α, IL-10, TGF-β, and β-actin in the Sham, MCAO, AS-IV, and NBP groups. **(B–E)** Densitometric quantification of IL-6, TNF-α, IL-10, and TGF-β, respectively, expressed as target protein/β-actin band intensity ratios. **(F–I)** ELISA measurements of IL-6, TNF-α, IL-10, and TGF-β, respectively, in brain tissue homogenates; cytokine levels are expressed as pg/mg protein. Data are presented as mean ± SD. For western blot analysis, n = 6 mice per group; for ELISA analysis, n = 5 mice per group. Brackets indicate comparisons between the indicated groups. *P < 0.05, **P < 0.01, ***P < 0.001, ****P < 0.0001.

### 3.3 Transcriptomic analysis reveals global gene-expression changes and immune-related functional alterations after AS-IV treatment

To explore mechanisms potentially underlying AS-IV-mediated protection, RNA-seq was performed on brain tissue from the Sham, MCAO, and AS-IV groups. Principal component analysis was performed to assess sample-level transcriptomic variation, and the corresponding PCA plot is provided in **Supplementary Figure S1**. Using q < 0.05 and fold change > 1.5 or < 1/1.5 as the differential-expression threshold, 147 DEGs were identified in the MCAO-versus-Sham comparison, including 129 upregulated and 18 downregulated genes, whereas 214 DEGs were identified in the AS-IV-versus-MCAO comparison, including 52 upregulated and 162 downregulated genes. Complete DEG lists for the two comparisons are provided in **Supplementary Tables S2** and **S3**, respectively. Intersecting the two DEG sets while requiring opposite directions of regulation yielded 37 common oppositely regulated genes, which were visualized across all nine samples in the heatmap shown in **Figure 3A**. Volcano plots further illustrated the global distribution of DEGs in the two comparisons, with representative members of the common oppositely regulated gene set annotated (**Figure 3B**). These data suggest that AS-IV intervention may partly modify MCAO-induced transcriptomic alterations in brain tissue.

**Figure 3:**
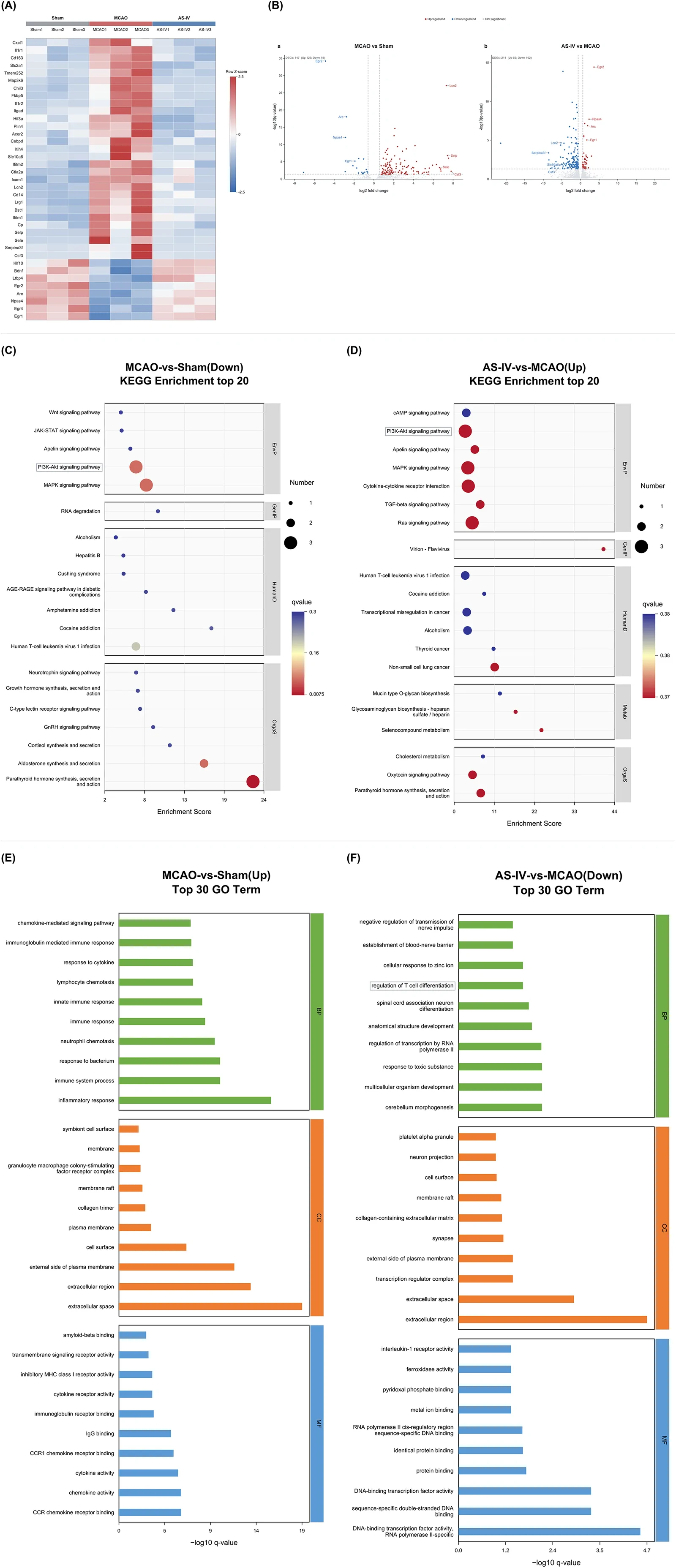
Transcriptomic analysis links PI3K/AKT-associated signaling and immune regulation to AS-IV responses after MCAO. **(A)** Heatmap showing the common oppositely regulated genes identified between the MCAO-versus-Sham and AS-IV-versus-MCAO comparisons across all nine RNA-seq samples. **(B)** Volcano plots showing the global distribution of differentially expressed genes in the MCAO-versus-Sham and AS-IV-versus-MCAO comparisons, with representative common oppositely regulated genes annotated. **(C)** KEGG enrichment analysis of genes downregulated in MCAO versus Sham, with the PI3K–Akt signaling pathway indicated. **(D)** KEGG enrichment analysis of genes upregulated in AS-IV versus MCAO, with the PI3K–Akt signaling pathway indicated. **(E)** GO enrichment analysis of genes upregulated in MCAO versus Sham, showing prominent immune- and inflammation-related biological processes. **(F)** GO enrichment analysis of genes downregulated in AS-IV versus MCAO, including the regulation of T-cell differentiation term. The T-cell differentiation-related term reflects transcriptional alteration of a regulatory process and should not be interpreted as indicating either enhanced or reduced T-cell/Treg differentiation. RNA-seq was performed using brain tissue collected on day 7 after MCAO from the Sham, MCAO, and AS-IV groups, with n = 3 biological replicates per group. Differentially expressed genes were identified using q-value < 0.05 and fold change > 1.5 for upregulated genes or fold change < 1/1.5 for downregulated genes. GO and KEGG over-representation analyses were performed using the corresponding GO- and KEGG-annotated background gene sets, and P values were adjusted for multiple testing using the Benjamini–Hochberg method. Enrichment significance in panels **(C–F)** is displayed using adjusted q-values. Transcriptomic analysis was based on the *Mus musculus* NCBI GRCm39 reference genome and the corresponding GO and KEGG annotations.

KEGG over-representation analysis identified the PI3K–Akt signaling pathway among the top-ranked pathways associated with genes downregulated in the MCAO group compared with the Sham group and genes upregulated in the AS-IV group compared with the MCAO group (**Figures 3C, D**). However, the enrichment did not consistently retain statistical significance after Benjamini–Hochberg correction, indicating that these results should be interpreted as exploratory pathway-level signals rather than definitive evidence of pathway activation. Complete KEGG enrichment results for upregulated and downregulated genes in both comparisons are provided in **Supplementary Tables S4**-**S7**.

GO enrichment analysis identified multiple immune- and inflammation-related biological processes after MCAO (**Figure 3E**). In the AS-IV-versus-MCAO comparison, genes downregulated after AS-IV treatment were enriched in several immune-related terms, including regulation of T-cell differentiation and interleukin-1 receptor activity (**Figure 3F**). Because “regulation of T-cell differentiation” represents a regulatory process rather than a direct measure of T-cell differentiation itself, its enrichment within the downregulated gene set should not be interpreted as indicating reduced or enhanced T-cell/Treg differentiation. Instead, these findings suggest that AS-IV was associated with transcriptional remodeling of immune- and T-cell-related regulatory programs. The direction and cellular consequences of these changes were therefore examined directly in subsequent Treg-related experiments. Complete GO enrichment results are provided in **Supplementary Tables S8**-**S11**.

To provide a less threshold-dependent assessment, ranked GSEA was additionally examined using the full gene-expression ranking. For the PI3K–Akt signaling pathway, nominal enrichment was observed in the MCAO-versus-Sham comparison (NES = 1.35, nominal P = 0.012), but this did not remain significant after FDR correction (FDR = 0.150). No significant enrichment was observed in the AS-IV-versus-MCAO comparison (NES = −1.03, nominal P = 0.381, FDR = 0.845). Complete GSEA results are provided in the **Supplementary Tables S12**, **S13**.

Taken together, the transcriptomic analyses identified broad immune-related transcriptional alterations and highlighted PI3K/AKT as a candidate signaling axis, but did not by themselves establish coordinated PI3K/AKT pathway activation by AS-IV. PI3K/AKT-associated protein changes and Treg-related immune responses were therefore examined using independent experimental approaches.

### 3.4 AS-IV modulates PI3K/AKT-associated signaling after MCAO

To further examine PI3K/AKT-associated signaling at the protein level, western blotting was used to measure PI3K and AKT phosphorylation. Compared with the Sham group, the MCAO group showed a lower p-PI3K/PI3K ratio, whereas AS-IV treatment increased this ratio relative to the MCAO group. The p-AKT/AKT ratio followed the same pattern (**Figures 4A–C**). These findings indicate that PI3K/AKT-associated phosphorylation was reduced after MCAO and increased after AS-IV treatment, providing independent protein-level evidence of PI3K/AKT-associated signaling alterations.

**Figure 4:**
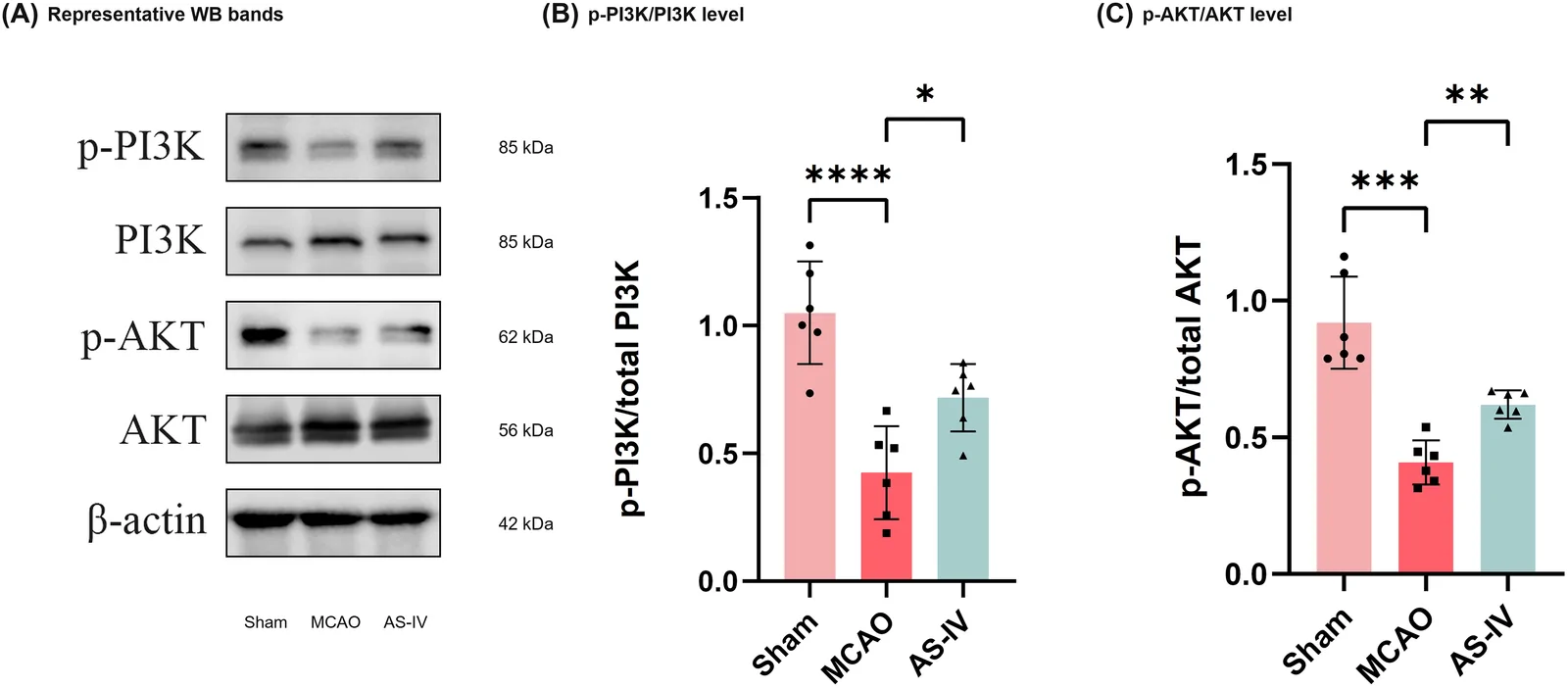
AS-IV regulates PI3K/AKT-associated signaling after MCAO. **(A)** Representative western blots of p-PI3K, PI3K, p-AKT, AKT, and β-actin in the Sham, MCAO, and AS-IV groups. **(B)** Densitometric quantification of the p-PI3K/PI3K ratio. **(C)** Densitometric quantification of the p-AKT/AKT ratio. Data are presented as mean ± SD (n = 6 mice per group). Brackets indicate comparisons between the indicated groups. *P < 0.05, **P < 0.01, ***P < 0.001, ****P < 0.0001.

### 3.5 AS-IV enhances Treg-related immune responses after MCAO

Because transcriptomic analysis identified alterations in immune regulatory and T-cell differentiation-related processes, without establishing the direction of Treg differentiation, Treg-related changes were subsequently examined directly in peripheral lymphoid organs and brain tissue. Flow cytometry was used to quantify CD4^+^CD25^+^FOXP3^+^ regulatory populations in the spleen and cervical lymph nodes, whereas immunofluorescence staining was used to assess CD3^+^FOXP3^+^ signals in brain tissue. Considering the potential influence of anti-CD25 intervention on CD25-based detection, CD4^+^FOXP3^+^ populations were additionally analyzed as a CD25-independent assessment of Treg-related changes. In the spleen, Treg proportions were mildly increased in the MCAO group compared with the Sham group and were further increased after AS-IV treatment (**Figures 5A–C**). Anti-CD25 intervention markedly reduced splenic Treg proportions, while the Anti-CD25+AS-IV group showed a partial recovery trend. Cervical lymph node results were generally consistent with splenic results. Compared with the MCAO group, the AS-IV group had a higher Treg proportion. Anti-CD25 intervention reduced Treg proportions, and the Anti-CD25+AS-IV group partially recovered compared with the Anti-CD25 group but remained lower than the AS-IV group (**Figures 5D, E**). To further validate the Treg-related changes independently of CD25 expression, CD4^+^FOXP3^+^ populations were additionally analyzed. Similar trends were observed among experimental groups (**Supplementary Figure S2**). In brain tissue, the proportion of CD3^+^FOXP3^+^ cells was increased in the AS-IV group compared with the MCAO group. Anti-CD25 intervention reduced the proportion of CD3^+^FOXP3^+^ cells, whereas the Anti-CD25+AS-IV group showed partial recovery compared with the Anti-CD25 group (**Figures 5F, G**). These results suggest that AS-IV increased peripheral Treg proportions and enhanced CD3^+^FOXP3^+^ signals in brain tissue after MCAO, while Anti-CD25 intervention attenuated these changes.

**Figure 5:**
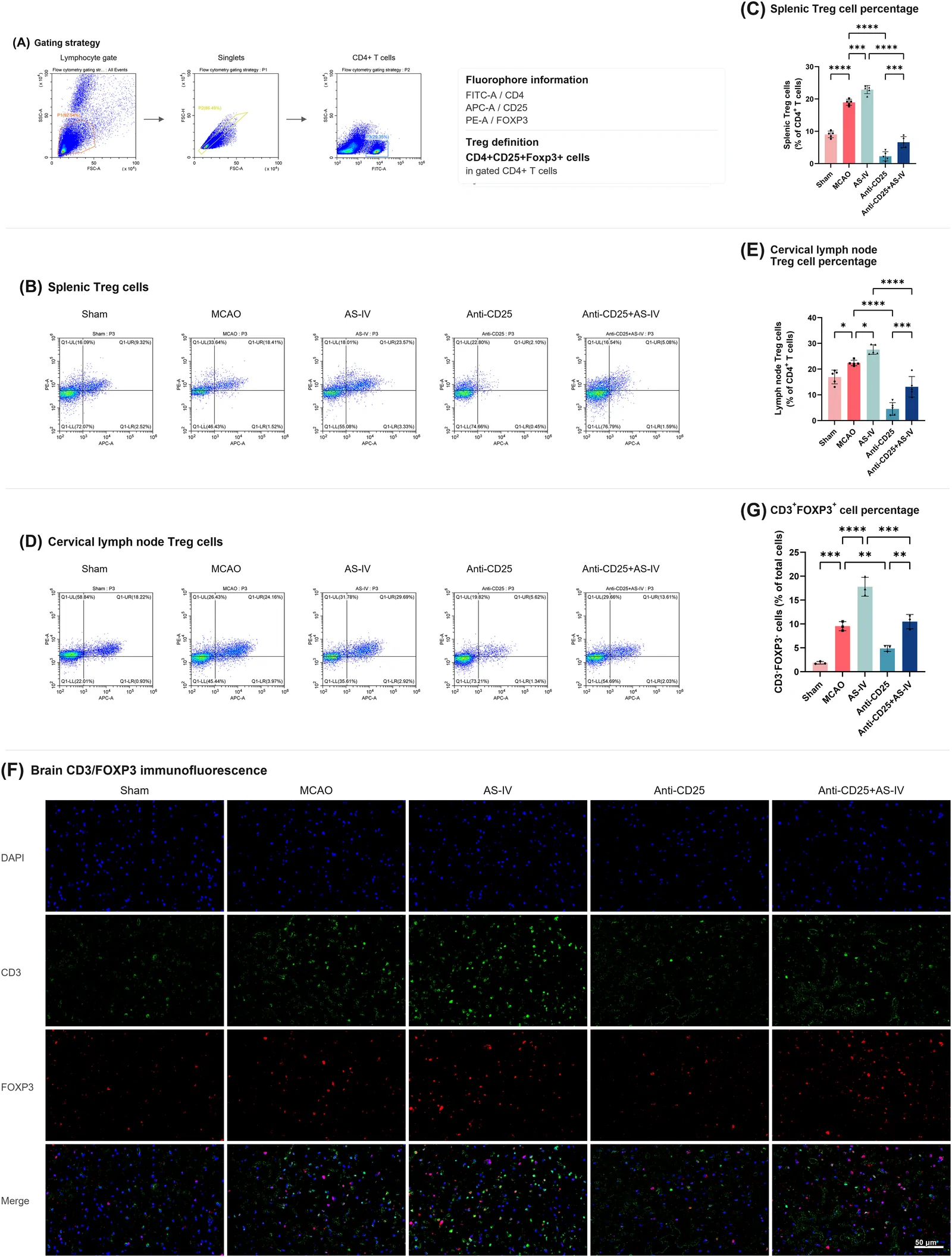
AS-IV enhances peripheral Treg-related immune responses and CD3^+^FOXP3^+^ signals in brain tissue after MCAO. **(A)** Gating strategy used to identify CD4^+^CD25^+^FOXP3^+^ Tregs. **(B, C)** Representative flow cytometry plots and quantification of splenic Tregs. **(D, E)** Representative flow cytometry plots and quantification of Tregs in cervical lymph nodes. **(F)** Representative DAPI, CD3, FOXP3, and merged immunofluorescence images of brain tissue from the Sham, MCAO, AS-IV, Anti-CD25, and Anti-CD25+AS-IV groups. **(G)** Quantification of CD3^+^FOXP3^+^ cells as a percentage of total DAPI^+^ cells. Scale bar, 50 μm. Data are presented as mean ± SD. For flow cytometry analysis, n = 5 mice per group; for immunofluorescence analysis, n = 3 mice per group. Three fields per mouse were analyzed and averaged to obtain one value for each animal. Brackets indicate comparisons between the indicated groups. *P < 0.05, **P < 0.01, ***P < 0.001, ****P < 0.0001.

### 3.6 Anti-CD25 intervention partially attenuates AS-IV-associated improvements in neurological deficits and ischemic brain injury after MCAO

To further determine whether Treg-related immune responses were associated with AS-IV-related improvements in neurological function and ischemic brain injury, these outcomes were additionally evaluated in the five-group mechanistic cohort. The mNSS was used to provide a more comprehensive assessment of neurological deficits in this cohort, as it evaluates multiple functional domains and allows a more detailed assessment of neurological outcome after Anti-CD25 intervention. Compared with the Sham group, mNSS scores were markedly increased in the MCAO group. AS-IV treatment significantly reduced mNSS scores compared with the MCAO group. Compared with Anti-CD25 intervention alone, combined Anti-CD25 and AS-IV treatment significantly reduced mNSS scores. Compared with AS-IV treatment alone, the Anti-CD25+AS-IV group showed a trend toward higher scores, although the difference was not statistically significant (**Figure 6C**). These findings suggest that attenuation of Treg-related immune responses was accompanied by worsening neurological deficits, whereas AS-IV retained a partial functional benefit under Anti-CD25 intervention.

**Figure 6:**
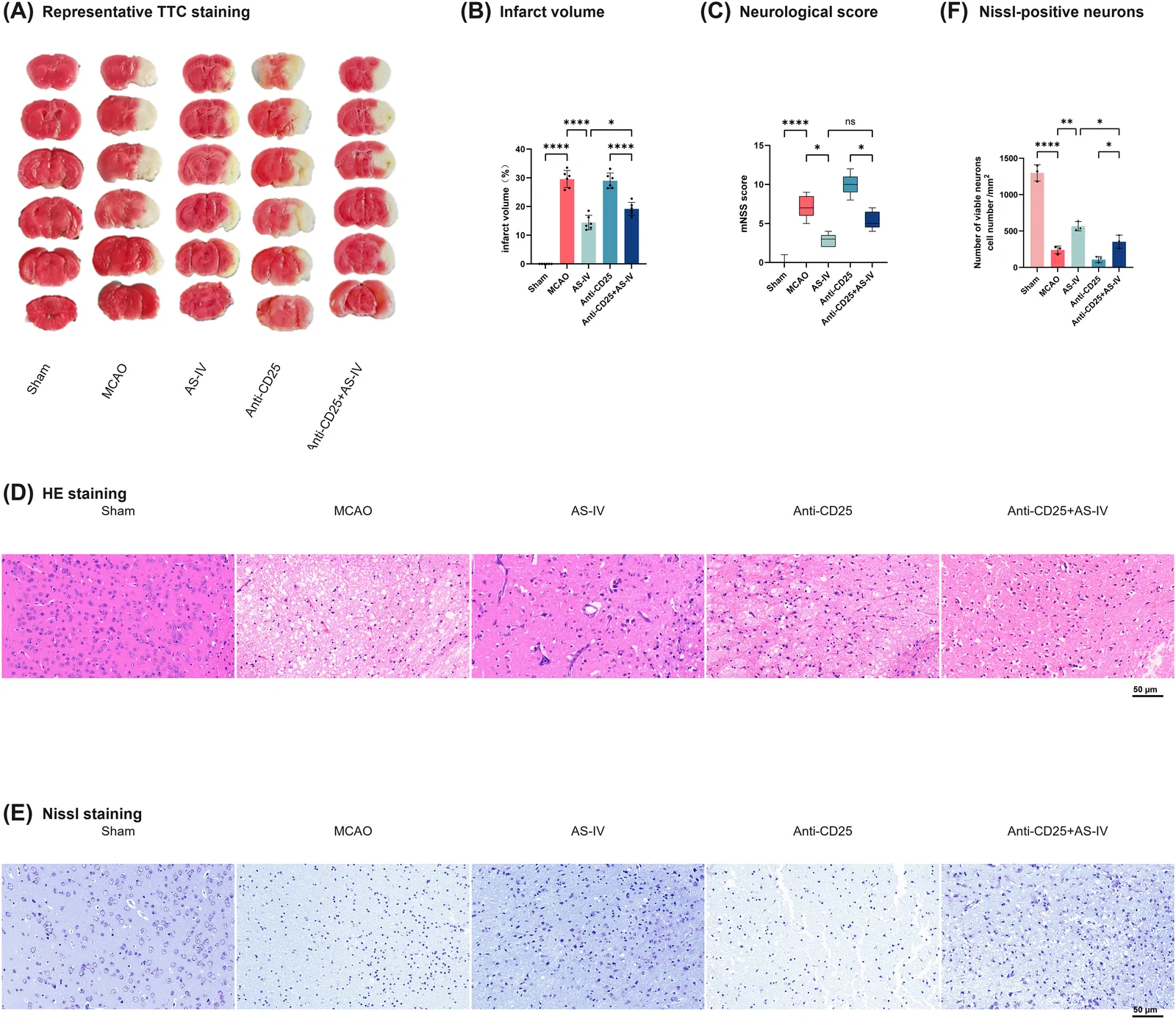
Anti-CD25 intervention partially attenuates AS-IV-associated improvements in neurological deficits and ischemic brain injury after MCAO. **(A)** Representative TTC-stained brain sections from the Sham, MCAO, AS-IV, Anti-CD25, and Anti-CD25+AS-IV groups. **(B)** Quantification of corrected cerebral infarct volume. **(C)** Modified Neurological Severity Score (mNSS) assessed 7 days after reperfusion. **(D)** Representative HE-stained brain sections. **(E)** Representative Nissl-stained brain sections. **(F)** Quantification of Nissl-positive neurons. Continuous data are presented as mean ± SD, whereas mNSS scores in **(C)** are presented as median with interquartile range (IQR). For mNSS assessment, n = 9 mice per group; for TTC analysis, n = 6 mice per group; for HE and Nissl staining, n = 3 mice per group. Brackets indicate comparisons between the indicated groups. *P < 0.05, **P < 0.01, ***P < 0.001, ****P < 0.0001.

TTC staining showed a marked increase in cerebral infarct volume in the MCAO group compared with the Sham group, whereas AS-IV significantly reduced infarct volume compared with MCAO. Compared with the AS-IV group, the Anti-CD25+AS-IV group showed a significant increase in infarct volume, while infarct volume was significantly lower in the Anti-CD25+AS-IV group than in the Anti-CD25 group (**Figures 6A, B**). HE staining further showed more pronounced ischemic histopathological changes in the MCAO and Anti-CD25 groups, whereas these changes were alleviated in the AS-IV and Anti-CD25+AS-IV groups (**Figure 6D**). Consistent with the TTC findings, Nissl staining showed a marked reduction in Nissl-positive neurons in the MCAO group compared with the Sham group, whereas AS-IV significantly increased neuronal preservation compared with MCAO. Compared with the AS-IV group, the Anti-CD25+AS-IV group showed a significant reduction in Nissl-positive neurons, while Nissl-positive neurons were significantly increased in the Anti-CD25+AS-IV group compared with the Anti-CD25 group (**Figures 6E, F**). Together, these findings indicate that Anti-CD25 intervention partially weakened the neuroprotective effects associated with AS-IV, whereas AS-IV retained partial protection under Anti-CD25 intervention.

### 3.7 Anti-CD25 partially attenuates the regulatory effects of AS-IV on inflammatory cytokine expression

To determine whether Treg-related immune responses were involved in AS-IV-mediated regulation of the neuroinflammatory microenvironment after MCAO, western blotting and ELISA were used to compare inflammatory cytokine levels across the five groups. Western blot analysis showed marked increases in IL-6 and TNF-α expression in the MCAO group compared with the Sham group. AS-IV treatment reduced IL-6 and TNF-α expression relative to the MCAO group. In the Anti-CD25+AS-IV group, IL-6 and TNF-α expression levels were higher than those in the AS-IV group but lower than those in the Anti-CD25 group (**Figures 7A–C**), indicating that Anti-CD25 intervention partially weakened but did not eliminate the anti-inflammatory regulatory effects associated with AS-IV. For the immunoregulatory cytokines IL-10 and TGF-β, AS-IV increased expression compared with the MCAO group (**Figures 7D, E**). Anti-CD25 intervention reduced IL-10 and TGF-β expression, whereas the Anti-CD25+AS-IV group showed partial recovery compared with the Anti-CD25 group. ELISA results were generally consistent with the western blot results. AS-IV reduced IL-6 and TNF-α levels and increased IL-10 and TGF-β levels compared with the MCAO group, whereas Anti-CD25 intervention on the basis of AS-IV treatment partially attenuated these changes (**Figures 7F–I**). These findings indicate that Treg-related immune responses may participate in AS-IV-mediated regulation of the neuroinflammatory microenvironment after MCAO.

**Figure 7:**
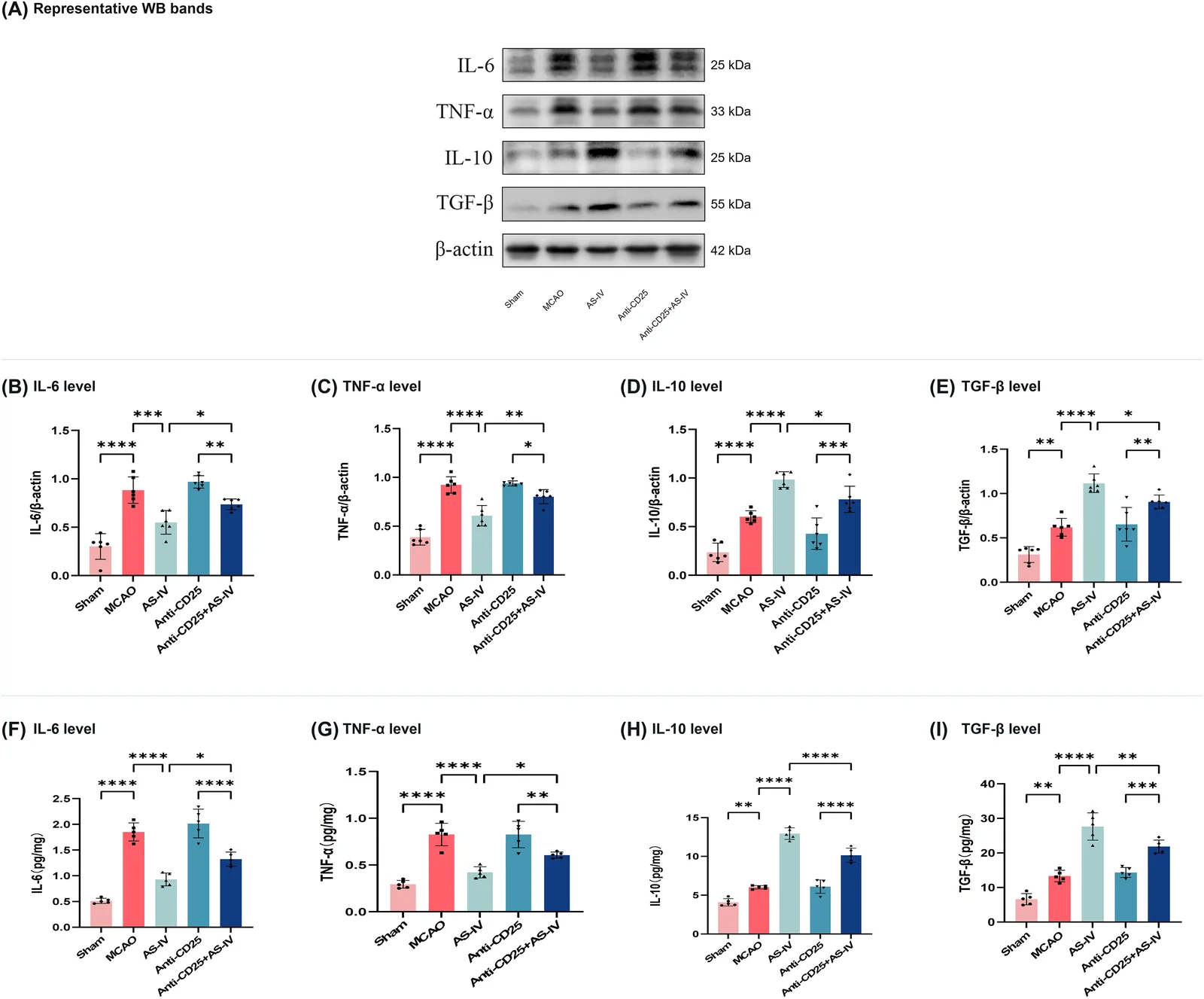
Anti-CD25 intervention partially attenuates the effects of AS-IV on inflammatory cytokine expression after MCAO. **(A)** Representative western blots of IL-6, TNF-α, IL-10, TGF-β, and β-actin in the Sham, MCAO, AS-IV, Anti-CD25, and Anti-CD25+AS-IV groups. **(B–E)** Densitometric quantification of IL-6, TNF-α, IL-10, and TGF-β, respectively, expressed as target protein/β-actin band intensity ratios. **(F–I)** ELISA measurements of IL-6, TNF-α, IL-10, and TGF-β, respectively, in brain tissue homogenates; cytokine levels are expressed as pg/mg protein. Data are presented as mean ± SD. For western blot analysis, n = 6 mice per group; for ELISA analysis, n = 5 mice per group. Brackets indicate comparisons between the indicated groups. *P < 0.05, **P < 0.01, ***P < 0.001, ****P < 0.0001.

### 3.8 AS-IV modulates CD86/CD206 associated brain myeloid phenotypic responses after MCAO

Flow cytometry and immunofluorescence staining were used to examine how AS-IV affected CD86/CD206-associated brain myeloid phenotypic responses in brain tissue from the five groups. The brain-tissue flow cytometry gating strategy is shown in **Figure 8A**. Flow cytometric analysis showed that the proportion of CD86^+^ cells within the CD11b^+^CD45^low/int population was increased in the MCAO group compared with the Sham group, whereas AS-IV treatment reduced this proportion (**Figures 8B, C**). After Anti-CD25 intervention, the proportion of CD86^+^ cells within this population increased, and the Anti-CD25+AS-IV group showed values between the AS-IV and Anti-CD25 groups. The proportion of CD206^+^ cells within the CD11b^+^CD45^low/int population increased after MCAO and increased further after AS-IV treatment. Anti-CD25 intervention reduced the proportion of CD206^+^ cells, whereas the Anti-CD25+AS-IV group showed partial recovery compared with the Anti-CD25 group (**Figures 8D, E**). These flow cytometry results suggest that AS-IV reduced CD86-associated and increased CD206-associated phenotypic responses within the CD11b^+^CD45^low/int brain myeloid population.

**Figure 8:**
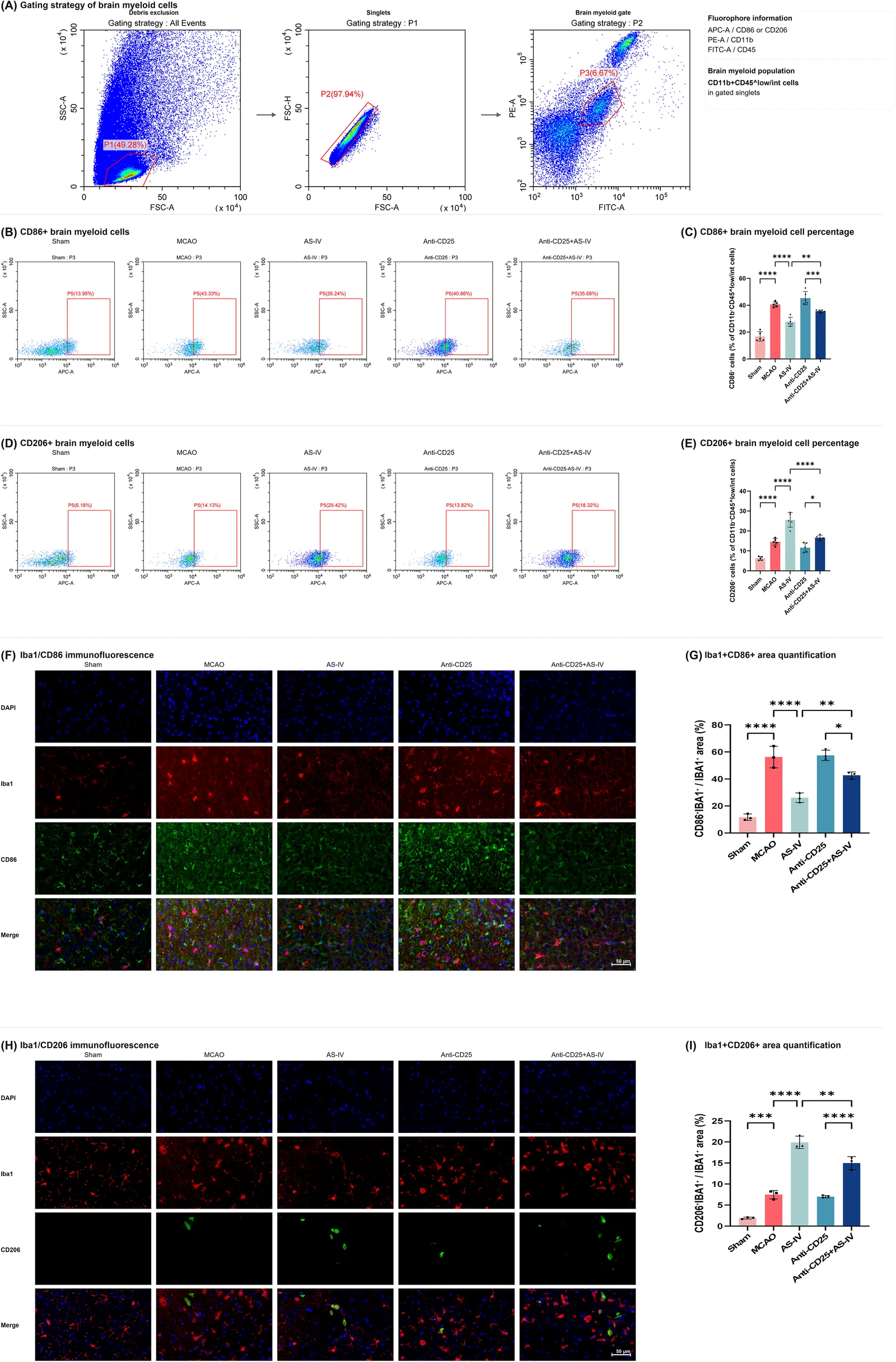
AS-IV regulates CD86/CD206-associated brain myeloid phenotypic responses after MCAO. **(A)** Flow cytometry gating strategy, including debris exclusion, singlet selection, and identification of the CD11b^+^CD45^low/int population. **(B, C)** Representative flow cytometry plots and quantification of CD86^+^ cells within the CD11b^+^CD45^low/int population. **(D, E)** Representative flow cytometry plots and quantification of CD206^+^ cells within the CD11b^+^CD45^low/int population. **(F, G)** Representative DAPI/Iba1/CD86 immunofluorescence images and quantification of Iba1^+^CD86^+^/Iba1 area (%). **(H, I)** Representative DAPI/Iba1/CD206 immunofluorescence images and quantification of Iba1^+^CD206^+^/Iba1 area (%). The groups included Sham, MCAO, AS-IV, Anti-CD25, and Anti-CD25+AS-IV. CD86^+^ and CD206^+^ cells were quantified as the percentage of marker-positive cells within the gated CD11b^+^CD45^low/int population. Scale bars, 50 μm. Data are presented as mean ± SD. For flow cytometry analysis, n = 5 mice per group; for immunofluorescence analysis, n = 3 mice per group. Three fields per mouse were analyzed and averaged to obtain one value for each animal. Brackets indicate comparisons between the indicated groups. *P < 0.05, **P < 0.01, ***P < 0.001, ****P < 0.0001.

Immunofluorescence staining supported the flow cytometry results. Iba1/CD86 double staining showed enhanced Iba1^+^CD86^+^ signals after MCAO, reduced signals after AS-IV treatment, and renewed increases after Anti-CD25 intervention (**Figures 8F, G**). Conversely, Iba1/CD206 double staining showed stronger Iba1^+^CD206^+^ signals in the AS-IV group, weaker signals after Anti-CD25 intervention, and partial recovery in the Anti-CD25+AS-IV group (**Figures 8H, I**). Together, these results indicate that AS-IV modulated CD86/CD206-associated brain myeloid phenotypic responses in the brain after MCAO. Anti-CD25 intervention attenuated this effect, suggesting that Treg-related immune responses may participate in AS-IV-associated regulation of brain myeloid phenotypic responses.

## 4 Discussion

Neuroinflammation contributes substantially to secondary injury and impaired neurological recovery after ischemic stroke (4). Although AS-IV has been reported to have anti-inflammatory, antioxidant, and neuroprotective activities (16, 20), it remains unclear whether its effects on the post-ischemic neuroinflammatory microenvironment involve Treg-related immune responses and brain myeloid phenotypic responses. In this study, AS-IV reduced cerebral infarct volume, showed a trend toward lower neurological deficit scores, and alleviated histopathological injury in MCAO mice. It also shifted the inflammatory cytokine profile toward lower pro-inflammatory cytokine expression and higher immunoregulatory cytokine expression. Transcriptomic analysis indicated that PI3K/AKT-associated signaling and immune regulatory processes may be involved in the effects of AS-IV. Subsequent experiments showed increased Treg proportions in the spleen and cervical lymph nodes and enhanced CD3^+^FOXP3^+^ signals in brain tissue after AS-IV treatment. In parallel, AS-IV modulated CD86/CD206-associated brain myeloid phenotypic responses in the ischemic brain. Anti-CD25 intervention reduced peripheral Treg proportions and CD3^+^FOXP3^+^ signals in brain tissue and partially attenuated the effects of AS-IV on neurological and brain injury outcomes, inflammatory cytokine expression, and brain myeloid phenotypic responses. Together, these findings suggest that AS-IV may modulate CD86/CD206-associated brain myeloid phenotypic responses and attenuate post-ischemic neuroinflammation, accompanied by alterations in Treg-related immune responses and PI3K/AKT-associated signaling.

Ischemic brain injury extends beyond the period of arterial occlusion. Post-ischemic inflammatory responses, peripheral immune cell infiltration, glial activation, and remodeling of the local inflammatory milieu can all contribute to secondary damage (4). We therefore evaluated AS-IV protection using neurological function assessment together with TTC, HE, and Nissl staining. AS-IV showed a trend toward lower neurological deficit scores, reduced the percentage of cerebral infarct volume, and alleviated structural damage and neuronal injury in brain tissue. These results establish an overall protective effect of AS-IV in the present MCAO model and support further analysis of its immunoinflammatory regulatory mechanisms.

Neuroinflammation is a central process in ischemic stroke progression. After ischemia, tissue hypoxia and disturbed energy metabolism can initiate inflammatory cascades, increase local pro-inflammatory cytokine release, and aggravate blood–brain barrier disruption, glial activation, and neuronal injury (21, 22). Immunoregulatory cytokines such as IL-10 and TGF-β help limit excessive inflammatory responses and support resolution of inflammation and restoration of the local microenvironment (23, 24). The balance between pro-inflammatory and immunoregulatory cytokines can therefore provide an index of the inflammatory status of the post-ischemic brain. In this study, IL-6 and TNF-α were markedly increased in the MCAO group, indicating a pronounced inflammatory response in brain tissue. IL-10 and TGF-β also increased to some extent, suggesting activation of endogenous immune regulatory responses after ischemic injury. Given the accompanying tissue injury and neurological deficits, this compensatory response was insufficient to halt inflammatory injury progression. AS-IV treatment decreased pro-inflammatory cytokine levels and increased immunoregulatory cytokine levels. Western blot and ELISA results showed generally consistent trends, indicating that AS-IV affected the broader cytokine expression pattern after MCAO rather than a single inflammatory mediator. These findings suggest that AS-IV-associated neuroprotection may involve improvement of the post-ischemic neuroinflammatory microenvironment.

Transcriptomic analysis was performed to explore molecular processes potentially associated with AS-IV intervention in the MCAO model. DEG-based over-representation analysis identified PI3K–Akt signaling among the higher-ranked candidate pathways, although pathway enrichment did not consistently remain significant after multiple-testing correction. Ranked GSEA, which was included to provide a less threshold-dependent assessment, similarly did not provide FDR-significant evidence for PI3K–Akt enrichment. Therefore, the transcriptomic data should not be interpreted as establishing PI3K/AKT pathway activation by AS-IV. Instead, they identified PI3K/AKT as a candidate signaling axis for subsequent protein-level examination. GO analyses also identified alterations in immune-, inflammatory-, and T-cell differentiation-related processes. Importantly, the occurrence of T-cell differentiation-related terms within the AS-IV-associated downregulated gene set does not indicate enhanced T-cell or Treg differentiation. These transcriptomic observations therefore served primarily as exploratory leads for subsequent evaluation of PI3K/AKT-associated signaling and Treg-related immune responses.

PI3K/AKT is an important signaling pathway involved in cell survival, inflammatory regulation, tissue repair, and microglial phenotypic regulation. During ischemic brain injury, this pathway may influence glial responses, inflammatory mediator expression, microglial phenotypic transitions, and neuronal repair processes (25). Previous studies have shown that PI3K/AKT-associated signaling can regulate CD86/CD206-defined microglial phenotypic responses after cerebral ischemia-reperfusion injury, accompanied by reduced expression of pro-inflammatory markers such as CD86 (26) and increased expression of anti-inflammatory or reparative markers such as CD206 (27). Here, the p-PI3K/PI3K and p-AKT/AKT ratios decreased after MCAO and increased after AS-IV treatment, indicating enhanced PI3K/AKT-associated phosphorylation following AS-IV intervention. Although PI3K–Akt pathway enrichment did not consistently retain statistical significance after multiple-testing correction, it remained among the higher-ranked candidate pathways in the transcriptomic analyses and showed a directionally relevant enrichment pattern. These transcriptomic findings therefore provided an exploratory basis for selecting PI3K/AKT for subsequent protein-level examination, and the western blot results provided additional support for PI3K/AKT-associated signaling alterations after AS-IV treatment. Together with previous evidence linking AS-IV-related neuroprotection to PI3K/AKT signaling regulation (28), these findings suggest that PI3K/AKT-associated signaling may participate in the effects of AS-IV on ischemic brain injury, inflammatory responses, and CD86/CD206-associated brain myeloid phenotypic responses. Several of the present observations are consistent with previous studies rather than representing entirely novel effects of AS-IV (16, 17, 19). Previous work has reported that AS-IV attenuates post-ischemic neuroinflammation, modulates PI3K/Akt-related signaling, and is associated with decreased CD86 and pro-inflammatory cytokines together with increased CD206, IL-10, and TGF-β. The present study therefore does not seek to establish these effects individually as novel findings. Instead, its conceptual advance lies in integrating Treg-related immune responses with these previously described neuroinflammatory and brain myeloid phenotypic changes *in vivo*. In particular, the anti-CD25 intervention provides additional evidence that Treg-related immune regulation may contribute to the broader neuroimmune responses associated with AS-IV treatment after MCAO.

Beyond intracellular signaling, transcriptomic analysis identified changes in immune regulatory, T-cell differentiation-related, and inflammatory processes after AS-IV intervention. These transcriptional changes did not by themselves establish enhanced Treg differentiation but provided a rationale for directly examining Treg-related immune responses. Tregs are a subset of CD4^+^ T cells with immunosuppressive and immune homeostasis-maintaining functions, and FOXP3 is closely related to their development and functional maintenance (29, 30). Tregs help restrict excessive inflammatory responses and maintain tissue immune balance. Previous studies have shown that Tregs form part of endogenous immune regulation after stroke and can limit excessive inflammation through immunoregulatory factors such as IL-10 and TGF-β. Conversely, impaired Treg-related immune responses may aggravate brain inflammation and neurological dysfunction (7). Studies also suggest that Tregs may participate in peripheral immune regulation and may enter the central nervous system after ischemia, where they can influence microglial activation states (31). We therefore examined Treg/FOXP3-related changes in both peripheral lymphoid organs and brain tissue to evaluate AS-IV effects on peripheral-central immunoinflammatory responses after ischemia. AS-IV increased Treg proportions in the spleen and cervical lymph nodes and enhanced CD3^+^FOXP3^+^ signals in brain tissue. These findings suggest that AS-IV-associated changes in local brain inflammation may be accompanied by enhanced Treg-related immune responses and altered peripheral–central immune communication after cerebral ischemia.

Anti-CD25 intervention was used to further assess the involvement of Treg-related immune responses in the effects of AS-IV. Anti-CD25 intervention reduced peripheral Treg proportions and CD3^+^FOXP3^+^ signals in brain tissue, indicating attenuation of Treg-related immune responses. Under this condition, IL-6 and TNF-α levels were higher in the Anti-CD25+AS-IV group than in the AS-IV group, whereas AS-IV-induced increases in IL-10 and TGF-β were partially suppressed. These findings indicate that Anti-CD25 intervention partially attenuated the regulatory effects of AS-IV on the inflammatory cytokine environment in the brain. Therefore, AS-IV-associated improvement of the inflammatory microenvironment after MCAO may not simply reflect direct suppression of pro-inflammatory cytokines. It may also be linked to enhanced Treg-related immune responses. This finding extends previous studies of the direct anti-inflammatory effects of AS-IV by suggesting that Treg-related immune regulation may represent an additional immunological component of its post-ischemic effects.

The additional neuroprotective outcomes assessed in the Anti-CD25 mechanistic cohort further support this interpretation. Anti-CD25 intervention was associated with poorer neurological and histopathological outcomes, whereas combined Anti-CD25 and AS-IV treatment partially improved mNSS scores, reduced cerebral infarction, and preserved neuronal morphology compared with Anti-CD25 intervention alone. These findings suggest that attenuation of Treg-related immune responses is accompanied not only by changes in the inflammatory microenvironment but also by worsening functional and structural brain injury, while AS-IV retains partial neuroprotective effects under Anti-CD25 intervention.

Microglia are resident immune cells in the brain and contribute to both inflammatory amplification and tissue repair after ischemic brain injury (32). After ischemia, microglia show heterogeneous activation states. CD86 is commonly used as a representative marker of pro-inflammatory M1-like phenotypic responses, whereas CD206 is often used as a marker associated with immunoregulatory and reparative M2-like phenotypic responses. Because myeloid activation states after ischemic stroke are heterogeneous and cannot be reduced to a binary M1/M2 framework, CD86 and CD206 were used as representative markers of inflammatory and immunoregulatory/reparative phenotypic responses, respectively (33). Within the gated CD11b^+^CD45^low/int population, the proportion of CD86^+^ cells increased after MCAO and decreased after AS-IV treatment, whereas the proportion of CD206^+^ cells was further increased after AS-IV treatment. These findings suggest that AS-IV modulates CD86/CD206-associated brain myeloid phenotypic responses after MCAO, with lower CD86-associated pro-inflammatory and higher CD206-associated immunoregulatory/reparative responses.

However, the CD11b^+^CD45^low/int gating strategy used in the present study may not completely distinguish resident microglia from infiltrating monocyte-derived macrophages after ischemic stroke, particularly because activated microglia may exhibit increased CD45 expression. In addition, Iba1 is expressed by both microglia and macrophage populations. Therefore, the CD86/CD206-associated changes observed in the present study are more appropriately interpreted as brain myeloid phenotypic responses rather than strictly microglia-specific alterations.

Anti-CD25 intervention was then used to examine whether Treg-related immune responses were involved in AS-IV-associated regulation of brain myeloid phenotypic responses. After Anti-CD25 intervention, the ability of AS-IV to reduce CD86-associated brain myeloid responses and enhance CD206-associated responses was partially attenuated. These results suggest that Treg-related immune responses may participate in the regulation of brain myeloid responses with AS-IV treatment. Together with the cytokine data, the findings support a model in which AS-IV enhances Treg-related immune regulation and increases immunoregulatory cytokines such as IL-10 and TGF-β. These changes may be associated with reduced pro-inflammatory cytokine release, lower CD86-associated brain myeloid phenotypic responses, and enhanced CD206-associated brain myeloid phenotypic responses. Thus, AS-IV-mediated regulation of neuroinflammation after MCAO may involve coordinated changes in Treg-related immune responses, inflammatory cytokine profiles, and brain myeloid phenotypic responses.

Based on these findings, we propose a potential model of AS-IV-associated neuroimmune regulation (**Figure 9**). Ischemic brain injury initiates inflammatory responses, increases CD86-associated brain myeloid responses and pro-inflammatory cytokine levels, and is accompanied by reduced PI3K/AKT-associated signaling. After AS-IV treatment, PI3K/AKT-associated phosphorylation is increased, peripheral Treg proportions and CD3^+^FOXP3^+^ signals in brain tissue are enhanced, CD86-associated brain myeloid phenotypic responses are reduced, CD206-associated brain myeloid phenotypic responses are increased, and inflammatory cytokine profiles are improved. Anti-CD25 intervention partially attenuated the regulatory effects of AS-IV on inflammatory cytokines and brain myeloid phenotypic responses, further suggesting that Treg-related immune responses may participate in the neuroprotective effects of AS-IV. This proposed model may help explain how AS-IV-associated neuroprotection is accompanied by coordinated changes in immune regulation, brain myeloid phenotypic responses, and the neuroinflammatory microenvironment.

**Figure 9:**
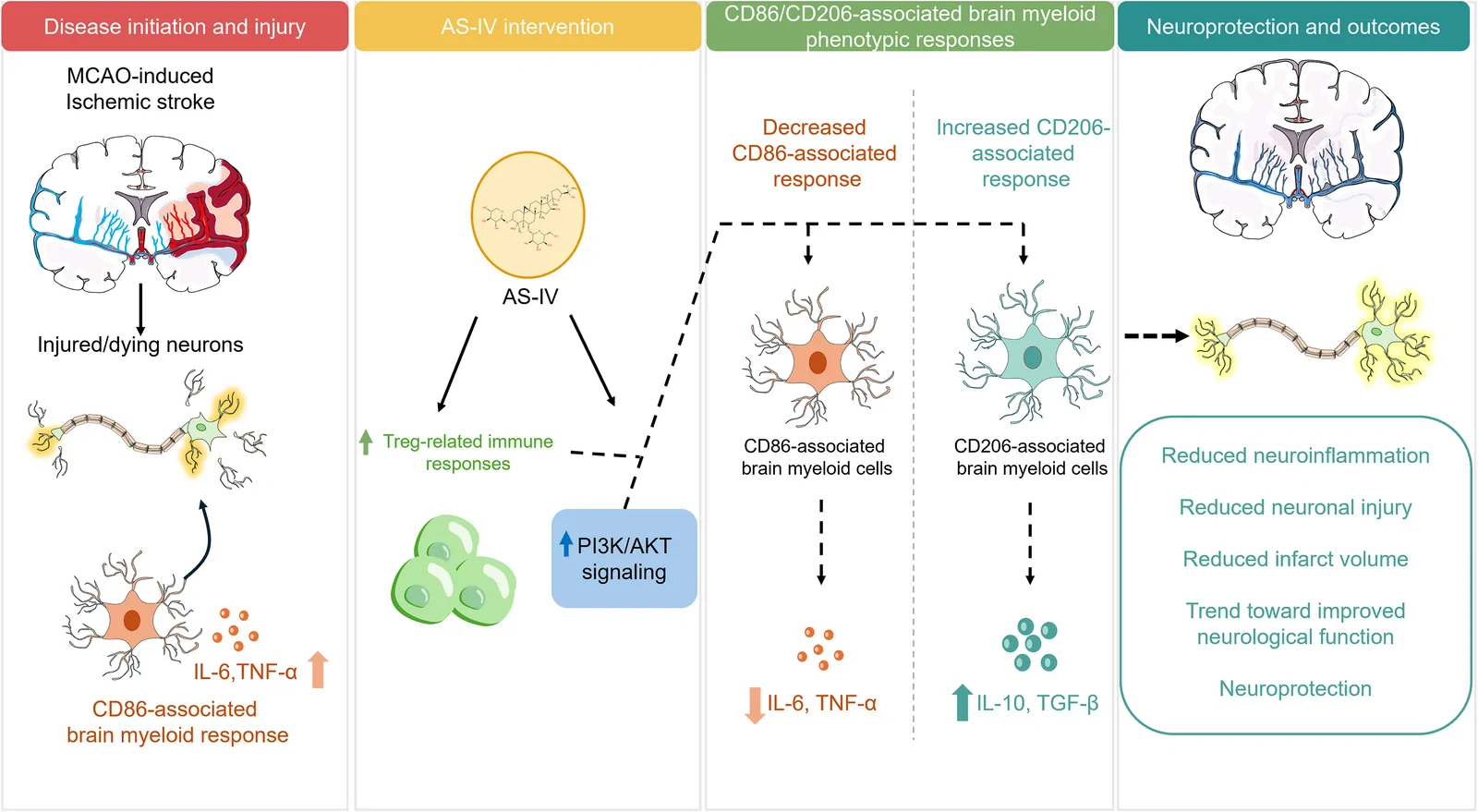
Proposed model of AS-IV-associated neuroimmune regulation after MCAO. MCAO-induced ischemic injury is associated with neuronal damage, enhanced CD86-associated brain myeloid responses, and increased IL-6 and TNF-α expression. AS-IV treatment is accompanied by enhanced Treg-related responses and increased PI3K/AKT-associated signaling, together with decreased CD86-associated and increased CD206-associated brain myeloid phenotypic responses. These changes are associated with reduced pro-inflammatory cytokines, increased immunoregulatory cytokines, reduced neuroinflammation and neuronal injury, decreased infarct volume, and a trend toward improved neurological function. Solid arrows indicate relationships directly supported by the present experimental findings, whereas dashed arrows indicate proposed mechanistic links that were not directly demonstrated in this study. CD86- and CD206-associated responses are depicted as parallel phenotypic changes and do not imply direct conversion between these populations.

This study has several limitations. First, although transcriptomic and western blot results suggest that PI3K/AKT-associated signaling may participate in the effects of AS-IV, PI3K/AKT pathway inhibitors or cell-specific interventions were not used. In addition, RNA-seq was performed on whole ischemic brain tissue with only three biological replicates per group. Moreover, PI3K–Akt pathway enrichment did not consistently retain statistical significance after multiple-testing correction, and ranked GSEA did not provide FDR-significant evidence of coordinated pathway enrichment. Therefore, the transcriptomic findings should be interpreted as exploratory rather than as definitive evidence of PI3K/AKT pathway activation. The observed immune-related and PI3K/AKT-associated transcriptional signatures may also partly reflect changes in cellular composition rather than cell-specific pathway activation. The necessity and cell-specific involvement of this pathway in AS-IV-associated neuroprotection therefore remain to be further investigated. Second, although anti-CD25 intervention attenuated Treg-related responses and CD4^+^FOXP3^+^ analysis showed consistent trends independent of CD25 expression, the specificity of anti-CD25-mediated modulation remains limited. CD25 is also expressed by activated conventional T cells, and potential interference between PC61-mediated CD25 targeting and subsequent CD25 detection cannot be completely excluded. Future studies using more Treg-specific approaches, such as Foxp3-based depletion models, adoptive transfer, or genetic strategies, may further clarify the causal contribution of Treg-related responses to the effects of AS-IV. Third, although CD3/FOXP3 double immunofluorescence staining was used to strengthen the identification of brain FOXP3-positive signals, this approach does not provide the same phenotypic resolution as brain flow cytometry or CD3/CD4/FOXP3 multi-marker analysis. Therefore, the brain immunofluorescence findings are described as CD3^+^FOXP3^+^ signals rather than as brain Treg cells. Brain flow cytometry or single-cell sequencing was not performed to define the source, proportion, and functional status of brain-infiltrating Treg populations. Fourth, the CD11b^+^CD45^low/int gating strategy and Iba1-based immunofluorescence used in this study cannot completely distinguish resident microglia from infiltrating monocyte-derived macrophages after ischemic stroke. Therefore, the observed CD86/CD206-associated changes should be interpreted as brain myeloid phenotypic responses rather than strictly microglia-specific effects. Future studies incorporating microglia-enriched markers such as TMEM119 or P2RY12 together with markers of infiltrating monocyte-derived populations may more precisely define the cellular origin of these responses. Fifth, although AS-IV treatment showed a trend toward lower Zea–Longa neurological deficit scores compared with the MCAO group, this difference did not reach statistical significance after nonparametric multiple-comparison correction. Therefore, the functional effects of AS-IV should be interpreted cautiously and warrant further validation in future studies using complementary neurological assessments. In addition, objective cerebral blood flow monitoring was not routinely performed to confirm successful arterial occlusion and reperfusion, and model inclusion was based primarily on the predefined postoperative neurological criterion. Future studies incorporating routine cerebral blood flow monitoring would provide more objective confirmation of successful MCAO and reperfusion. Finally, whether Treg-related immune responses directly or indirectly regulate brain myeloid phenotypic responses, and how IL-10, TGF-β, or PI3K/AKT-associated signaling may connect these processes, requires further investigation using *in vitro* co-culture, blocking experiments, and cell-specific validation.

## 5 Conclusion

In conclusion, this study shows that AS-IV reduces cerebral infarct injury, shows a trend toward improvement in neurological deficit scores, and alleviates neuroinflammation after MCAO. The effects of AS-IV may extend beyond suppression of a single inflammatory cytokine and may involve multilayered immunoinflammatory regulation, including PI3K/AKT-associated signaling, Treg-related immune regulation, and CD86/CD206-associated brain myeloid phenotypic responses. These findings provide experimental evidence supporting AS-IV as a potential intervention for post-ischemic neuroinflammation and provide a basis for further investigation of Treg-related regulation of brain myeloid phenotypic responses in ischemic brain injury.

## Data Availability

The datasets presented in this study can be found in online repositories. The names of the repository/repositories and accession number(s) can be found in the article/**Supplementary Material**.
