# Supplementary material for: Astragaloside IV attenuates post-ischemic neuroinflammation associated with Treg-related regulation of CD86/CD206-associated brain myeloid phenotypic responses and PI3K/AKT signaling alterations
Source: Front Immunol. 2026 Oct 1;17:1939189. doi: 10.3389/fimmu.2026.1939189 (PMC13668508; doi:10.3389/fimmu.2026.1939189)
Supplement: Supplementary file 1 [file DataSheet1.zip › supplementary materials/Supplementary material images.doc]

**Supplementary material images**

**Supplementary Figure S1. Principal component analysis of RNA-seq samples.**
Principal component analysis (PCA) of all nine RNA-seq samples from the Sham, MCAO, and AS-IV groups, with three biological replicates per group.

**Supplementary Figure S2. CD25-independent flow cytometric assessment of Treg-related populations.**
Representative flow cytometry plots and quantification of CD4⁺FOXP3⁺ populations analyzed independently of CD25 expression in the indicated experimental groups.
